# Dr. England on Disorders of the Kidneys

**Published:** 1830-04-01

**Authors:** 


### XVII.

### Observations on those Functional Disorders of the Kidneys which give rise to the Formation of Urinary Calculi; with Remarks on their frequency in the County of Norfolk. By William England, M.D.

Dr. England is evidently a very young physician; but, in this little Essay, he offers unequivocal evidence of a good education—of a respectable acquaintance with ancient and modern authors—and with a considerable talent for observation. All these he has turned to a fair account in the work before us. For reasons not very difficult to be divined, we shall confine our notice of Dr. E.'s book to those sections which result from his actual observations, passing over those portions in which it is natural for a young author to "shew his book-learnt skill."

We believe, with Dr. E., that the formation of urinary calculi is not to be explained on chemical principles. We know, indeed, that in a healthy state, the kidneys secrete healthy urine; and when the renal circulation and secretion are deranged, we shall find lithic, oxalic, phosphoric, and muriatic acids in excess, in combination with lime, ammonia, soda, and magnesia. We know further, that in many or most disorders of the digestive organs, the urine becomes turbid and loaded with unnatural, or at least unhealthy products; and therefore it is highly probable that calculous formations are connected with, if not dependent on, disorder of the digestive function, inducing deranged secretion in the urinary apparatus. This seems to be the opinion of our author, and in the 5th section of his work he takes up the subject of diet and habits of life, as exciting causes of renal diseases. The diet of the French, he remarks, renders them more obnoxious to some diseases, and less so to others. The weak and acid wines, the cider, and the small-beer, which form the principal beverages of the inhabitants—in conjunction with the quantities of fruit and vegetables which they devour, dispose them to diarrhœa, in consequence of which M. Broussais has found less difficulty in establishing his doctrine of "gastro-enterite," than he would have found in other countries. Those, indeed, who have had opportunities of observing the hecatombs of trash, both animal and vegetable, which are daily gorged by the French, must wonder how they ever came to be considered an abstemious people. They are the greatest gluttons on the face of this earth—and we have no hesitation in averring that they devour nearly double as much as their English neighbours. But of that we shall say more in some other place.

In Ireland, and in many districts of Scotland, potatoes, oatmeal, and milk, form the chief items in the culinary list, and bowel-complaints are common among them. But Dr. E. thinks, and probably with reason, that this diet is, upon the whole, "more nutritious, and more capable of preventing the occurrence of deranged functions, than the aliment upon which the inhabitants of this district (Norfolk) chiefly subsist." The mild and unirritating qualities of this food, he believes, precludes the development of scrofula and consequently of phthisis—diseases which he avers are, upon the aggregate, much less frequent in Ireland than in England. But if the Irish, from the more genial climate of their island, and the unirritating quality of their food, be less disposed to calculous complaints, they are more liable to hepatic affections than their English neighbours — probably from their partiality to the "mountain dew." Still Dr. E. believes that the "poteen" of Ireland, and the "glenlevet" of Scotland, are much less injurious to the constitution than British gin and brandy. Whisky, he avers, has a tendency to relax the bowels—the other spirits to constipate them. As it appears that calculus is much more prevalent in Norfolk than in any other county of England—and infinitely more so than in Ireland or Scotland, our author, who is a Norfolk man, has taken pains to investigate the modes of life and the diet of his countrymen, with the view of accounting for this lithic diathesis.

Norfolk is distinguished for opulence and industry, though the soil is, in many tracts very inferior, and was, not long ago, little more than barren sandy wastes. The working classes subsist almost wholly on farinaceous food—wheat-bread made from flour of as good a quality as that generally used by the higher ranks of society. It is, however, eaten fresh from the oven, when it is more indigestible, and more irritating to the stomach. This bread then forms the breakfast and dinner of the labourer, and is generally washed down with water or tea, milk being scarce. The toils of the day being over, the supper becomes the principal meal. This is "constructed of the same material (wheat-flour,) but made into a culinary preparation little known in other parts of England, Suffolk excepted. This is the Norfolk dumpling, formed by adding yeast or ferment to flour, and worked up into a kind of paste. The panary fermentation is then allowed to take place, and the dough is laid a rising (as it is called) for a short time. It is then boiled for about twenty minutes, if the palate of the consumer prefer a light dumpling—or half an hour, if a heavy dumpling be the favourite. Only a comparatively small proportion of potatoes or other vegetables is combined with this farinaceous diet, and the drink is water, or bad beer from the public house." Dr. E. avers that no alimentary preparation is more indigestible or irritating to the stomach and bowels than the Norfolk dumpling. Norfolk being a corn county, very few cows are kept, and consequently the labouring classes have very little milk or butter. The author also remarks that the labours of harvest, of threshing the corn, and of various other domestic avocations in this great agricultural county, throw an undue action on the lumbar muscles, and nothing is more common than to hear them complain of strains in the loins. This over-exertion of the lumbar muscles may, he thinks, disturb the functions of the kidneys, and assist in leading to lithic secretions and concretions. Climate next comes to be investigated by Dr. England, and he conceives that the climate of Ireland is as much more genial to health than that of Norfolk, as the immunity of the Irish from calculous affections is superior to that of the inhabitants of the said county. Passing over some observations on medicinal agents in calculous diseases, we shall give Dr. England's general conclusions, containing the pith and marrow of the publication.

SUMMARY CONCLUSIONS.

"1. That the proximate cause of calculous disease is, a certain disordered condition of the capillary vessels of the kidneys, which organs, instead of eliminating from the arterial blood sent to them, the natural secretions, are found to secrete a fluid, the constituent principles of which are different to those of healthy urine.

"2. That the circulation of the blood in these vessels becomes disturbed by the application of morbid stimuli, internally to the mucous membrane of the alimentary canal, or externally to the cutaneous system.

"3. Even in a state of health the constitution of the urine is perpetually changing, owing to the varied irritation of unnatural stimuli, applied externally or internally.

"4. A very frequent cause of calculous disease is, the presence of bad alimentary substances in the stomach and intestines, the sentient surface of which having become morbidly irritable; this irritation is sympathetically conveyed by nervous transmission to the capillary vessels of the kidneys.

"5. The impression of cold disturbing the functions of the skin, and destroying the balance of the circulation, may induce in one constitution dropsy, in another diabetes, and in a third calculous disease.

"6. The inhabitants of those countries, where calculous diseases are most prevalent, are continually liable to functional disorders of the kidneys, from the use of an irritating diet, and from exposure to a cold climate.

"7. The general diet of the great mass of the inhabitants of the county of Norfolk, though composed chiefly of food prepared from wheaten flour, is, owing to the alimentary form in which it is consumed, much more likely to induce morbid irritation, than the diet of the same classes of people in Ireland and Scotland.

"8. Certain physical characters of the earth's surface, together with the peculiarity of its geographical situation, must always render the county of Norfolk more liable to ungenial vicissitudes of climate than other districts, in which calculous diseases are infrequent.

"9. That wherever phthisis pulmonalis and scrophula are less endemic, calculous diseases are likewise extremely rare.

"10. In hot climates, where the stimulus of heat retains on the surface of the human body a large proportion of the sanguineous fluid, the generation of urinary calculi is almost unknown: the exclusive use of a vegetable diet may probably contribute to their non-formation.

"11. That although alkaline and acid remedies seem to produce their effect by acting chemically upon the constitution of the urine, it is probable that they act likewise as antacids and tonics, by restoring the functional condition of the stomach, and consequently improving the composition of the blood.

"12. That sedatives and counter-irritants act by their stimulus being conveyed through nervous transmission to the disordered renal capillaries, and removing the morbid condition of those vessels, during the existence of which urinary calculi are generated."

We hope Dr. England will prosecute not only this but many other medical inquiries, for which he is evidently well calculated by natural talent and acquired knowledge.

---

